# Influence of Thermal Annealing on Mechanical and Optical Property of SiO_2_ Film Produced by ALD

**DOI:** 10.3390/ma17020470

**Published:** 2024-01-19

**Authors:** Xintao Zhi, Xiaopeng Li, Songmei Yuan, Dasen Wang, Kehong Wang

**Affiliations:** 1 School of Mechanical Engineering, Nanjing University of Science and Technology, Nanjing 210094, China; zhiweiwei413@163.com (X.Z.); yuansm@buaa.edu.cn (S.Y.); wds9059@163.com (D.W.); 2 School of Materials Science and Engineering, Nanjing University of Science and Technology, Nanjing 210094, China

**Keywords:** film, thermal annealing, SiO_2_, spectrum, structure, property

## Abstract

The application range of fused silica optical components can be expanded and the cost of fused silica components can be reduced by depositing the same material film on fused silica substrate. However, due to the different manufacturing process, the performance of ALD SiO_2_ film is lower than that of fused silica substrate, which also limits the use of this process. In this paper, ALD SiO_2_ film with different thicknesses were deposited, and then the structure and properties were tested. Finally, the ALD SiO_2_ film was treated via the annealing process. Transmission electron microscopy (TEM) showed that the ALD SiO_2_ film had good compactness and substrate adhesion. The Raman spectra showed that the ALD SiO_2_ film and substrate had the same structure, with only slight differences. The XRD pattern showed that ALD-fused silica did not crystallize before or after annealing. The infrared spectra showed that there was an obvious Si-OH defect in the ALD SiO_2_ film. The laser damage showed that the ALD SiO_2_ film had a much lower damage threshold than the fused silica substrate. The nanoindentation showed that the mechanical properties of the ALD SiO_2_ film were much lower than those of the fused silica substrate. After a low-temperature annealing treatment, the ALD SiO_2_ film Si-OH defect was reduced, the ALD SiO_2_ film four-member ring content was increased, the elastic modulus of the ALD SiO_2_ film was increased from 45.025 GPa to 68.025 GPa, the hardness was increased from 5.240 GPa to 9.528 GPa, and the ALD SiO_2_ film damage threshold was decreased from 5.5 J/cm^2^ to 1.3 J/cm^2^.

## 1. Introduction

Current global concerns about energy resources are seeing a shift towards sustainable energy generation technologies [1]. Inertial fusion energy (IFE) is a new type of energy that obtains clean deuterium tritium (DT) fusion energy based on inertial confinement fusion (ICF) and has commercial application value. The drive device of laser inertial confinement nuclear fusion is a large and complex optical system that requires a large number of high-quality optical components. Fused silica is the amorphous state of silicon dioxide; has excellent heat resistance, with a melting point temperature as high as 1730 °C; can work at a high temperature of 1450 °C for a short time; has excellent transmittance in far ultraviolet light, visible light, and near infrared light; has higher mechanical properties than ordinary glass; and has a manufacturing process. Because of these excellent properties of fused silica, it is often used as a basic material for optical components [2]. Taking the National Ignition Facility (NIF) [3,4] built in the United States in 2009 as an example, there are 1728 fused silica windows and lenses in the diameter range of 0.5 to 1.0 m and 192 fused silica gratings and shields [5,6]. The Laser Megajoule (LMJ) device in France and the Shen Guang III series device constructed in China also use a large number of fused silica components. The most common method for producing fused silica workpieces is grinding [7]. Due to the high hardness and low fracture toughness, fused silica is removed in brittle mode [8] in the grinding process. Crack propagation is the main way to remove fused silica. Because the median crack is deep inside the fused silica, it cannot be removed during the grinding process. This leads to the inevitable existence of micro-cracks on the surface of the fused silica component. These micro-cracks can cause the enhancement of light intensity, which in turn causes the components to produce a large number of damage points of varying sizes under the laser density below its inherent damage threshold [9,10]. When the fused silica is polished, the cutting depth is very small, the cracks are also very small, and the defects are few. In order to reduce the surface defects of fused silica components, polishing and other precision processes are often used to manufacture fused silica components [11,12], which leads to long manufacturing cycles and high processing costs. Atomic layer deposition (ALD) is a coating technique based on the sequential reaction of gaseous reactants limited to the solid surface of the substrate. As a result, the ALD process can manufacture optical film with ultra-high uniformity and excellent thickness accuracy in local and large areas.

For the first few years, the precursor to SiO_2_ in the ALD process was an inorganic silicon precursor (SiCl_3_H, SiCl_4_, etc.) [13,14], but the reaction efficiency was low, impurities such as chlorine could not be avoided, and the film performance was poor. Due to the advantages of the ALD process, people have carried out extensive research on Si precursors for ALD SiO_2_. Then, there are different kinds of amino silanes, such as tris-dimethylaminosilane (SiH[N(CH_3_)_2_]_3_, TDMAS), bisdiethylaminosilane (SiH_2_[N(C_2_H_5_)_2_]_2_, BDEAS), bis-dimethylaminosilane (SiH_2_[N(CH_3_)_2_]_2_, BDMAS), bis-tert-butylaminosilane (SiH_2_[NH(C_4_H_9_)]_2_, BTBAS), etc. [15,16]. Deposition of silica over a wide temperature range has been achieved.

The ALD process is used to deposit SiO_2_ on fused silica substrate, which can achieve consistency in the film and the substrate material. It can fill the local and overall defects formed by grinding the fused silica components. This can improve its surface quality, reduce manufacturing difficulty and manufacturing costs, and reduce processing time. The service life of the fused silica optical components can also be extended by filling the damage pit of the fused silica components. Through the annealing process of heating, holding, and cooling, it can reduce material stress, reduce material defects, and improve material properties and has a wide range of applications in metal and non-metal fields. After annealing, the structure of fused silica relaxes for a long time and the internal stress and structural inhomogeneity of fused silica can be eliminated so that fused silica with better performance can be obtained.

Recently, thermal ALD deposition was carried out at substrate temperatures of 250–350 °C using commercial AP-LTO^®^ 330 (Air products &Chemicals Inc., Espoo, Finland), BTBAS (bis(tertiary-butylamino) silane), and 3DMAS (tris(dimethylamino) silane) as silicon precursors and O_3_ as an oxygen source. The deposition rate at different temperatures was studied. The adhesion properties between SiO_2_ and Si were investigated using scratch adhesion testing, with the results indicating that the adhesion for coatings on Si was good [17]. SiO_2_ thin film was successfully grown using tris[dimethylamino] silane (3DMAS) and bis[diethylamino]silane (BDEAS) with plasma activated oxygen as precursors, and the amorphous SiO_2_ film showed very low optical losses within a spectral range of 200 nm to 1100 nm. The film was optically homogeneous and possess good scalability of the film thickness [18]. Lately, the absorption of ALD SiO_2_ film at 355 nm measured by laser calorimeter has varied linearly with the film thickness. Fourier transform infrared (FTIR) spectra confirmed the presence of point defects in ALD SiO_2_ film, including non-bridging oxygen atoms and residual OH groups. ALD films were conditioned with a sub-nanosecond ultraviolet laser, and the damage threshold was increased [19]. HfO_2_/SiO_2_ periodic multilayer high-reflection mirrors were prepared with a reactive electron beam evaporation technique. Then, the deposited mirrors were annealed in the temperature range from 300 °C to 500 °C. Crystallinity and grain size (HfO_2_) increased upon annealing. The laser-induced damage threshold (LIDT) increased from 44.1 J/cm^2^ to 77.6 J/cm^2^ with annealing up to 400 °C [20]. Ta_2_O_5_ film was prepared on BK7 substrates with conventional electron beam evaporation deposition. The effects of the protective SiO_2_ layers and annealing on the laser-induced damage threshold (LIDT) of the films were investigated. Annealing was effective at decreasing the microdefect density and the absorption of the films. Moreover, the combination of the protective SiO_2_ layer and annealing maximized the LIDT of the Ta_2_O_5_ film [21]. At the same time, annealing of the film improved the damage threshold of HfO_2_ under near-infrared laser irradiation [22,23].

Previous studies have shown that SiO_2_ films prepared by ALD process have good light transmission and substrate-binding force. However, the difference in the ring structure and vibration form of the ALD SiO_2_ from the substrate, the hardness and elastic modulus from the substrate, and the effect of heat treatment on the molecular structure and mechanical properties of ALD SiO_2_ on fused silica substrate have not been extensively studied.

Annealing coatings is an effective method to improve their properties [24]. In this study, ALD SiO_2_ films of different thicknesses were deposited on fused silica substrate, and the same process was used to deposit SiO_2_ on silicon wafer substrate. We first studied the relationship between ALD SiO_2_ thickness and molecular structure, laser damage threshold, and mechanical properties. Secondly, we treated ALD SiO_2_ film on fused silica substrate with the annealing process. Thirdly, we studied the effect of temperature on the structure and properties of ALD SiO_2_ film.

## 2. Experimental Process

### 2.1. Sample Preparation

Fused silica substrate with a surface roughness of less than 2 nm (RMS) was polished before deposition. The substrate thickness was 2 mm. The silicon wafer substrate was polished before deposition and had a surface roughness of less than 2 nm (RMS). Prior to deposition, all substrates were ultrasonically cleaned with a mixture of deionized water and ethanol. Then, the fused silica substrate was chemically etched in a 5% hydrofluoric acid solution for 80 min to remove absorbed impurities (Ce, Fe, etc.) and passivation [25]. The substrate was then rinsed with deionized water to clean the surface. By measuring the height of the corroded part from the original surface, the corrosion depth was calculated to be about 4 μm. In beneq TFS 500, using BTBAS as a Si precursor and O_3_ as an oxygen source, a single SiO_2_ film was deposited on the previously prepared fused silica substrate and silica wafer substrates via the thermal ALD method at substrate temperatures of 300 °C. Per the typical ALD cycle, the first step was filling it with BTBAS for 0.5 s, the second step was filling it with nitrogen for 3 s, the third step was filling it with O_3_ for 2 s, and the last step was filling it with nitrogen for 3 s. The film thickness and growth rate of the deposited SiO_2_ were measured via spectroscopic ellipsometry. In our experiment, the growth rate of ALD SiO_2_ was 0.1 nm/cycle. In a super-clean room, samples were placed in Petri dishes and wrapped in tinfoil and then annealed in a box-type resistance furnace (Model: KXL-1200X, Hefei, China). The samples were heated at rate of 10 °C/min, then held for 1 h, and last, cooled to normal temperature in the furnace.

### 2.2. Characterization

The microstructure of the ALD SiO_2_ film sample on the silicon wafer substrate was observed with a spherical transmission electron microscope (model: Titan G2 60-300, FEI Eindhoven, The Netherlands). The microstructure of the ALD SiO_2_ film on the fused silica substrate was observed via X-ray diffraction (model: Bruker-AXS D8 Advance, Bruker, Karlsruhe, Germany). A laser damage test platform (355 nm@10 ns) was used to test the ALD SiO_2_ film on the fused silica substrate via the R-on-1 method, and the laser damage threshold of the ALD SiO_2_ film on the fused silica substrate before and after heat treatment was studied. A Fourier transform infrared (FTIR) spectrometer (model: NICOLETIS10, Thermo America, Medley, FL, USA) was used to measure the infrared spectra of the ALD SiO_2_ film and the fused silica substrate in order to study the structure and defects of the ALD SiO_2_ film before and after heat treatment. Raman spectrometers (model: Aramis, HORIBA H.J.Y Company, Loos, France) were used to measure the Raman spectra of the ALD SiO_2_ film and the fused silica in order to study the structure of the ALD SiO_2_ film before and after heat treatment. A nano-indentation instrument (model: Keysight G200, Keysight Technologies, Inc. Santa Rosa, CA, USA) was used to measure the mechanical property in order to study the hardness and the elastic modulus of the ALD SiO_2_ film before and after heat treatment.

## 3. Experimental Results and Analysis

### 3.1. ALD SiO_2_ Structure and Properties

#### 3.1.1. ALD SiO_2_ Structure

**Figure 1:**
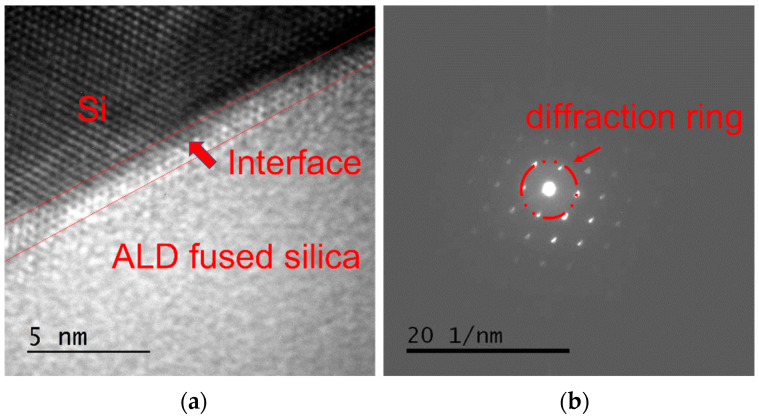
Transmission electron microscopy (TEM) photo of ALD SiO_2_ on silicon wafer substrate. (**a**) Lattice image of film interface and (**b**) electron diffraction pattern at the interface.

Transmission electron microscopy [26] can be used to observe the crystal structure of a sample and is commonly used to study the structure of thin film [27]. ALD SiO_2_ film on silicon wafer substrate was studied via transmission electron microscopy, and the images obtained are shown in Figure 1. Periodic arrangement of atoms in crystals can yield regular dark field images. It can be seen in Figure 1a that the ALD SiO_2_ film had a clear interface with the substrate, and the interface width was about 1.5 nm, which was close to the roughness of the silicon wafer. There were no obvious micro-defects such as holes or cracks at the interface, indicating that the adhesion of the atomic layer deposition was excellent. The accelerated and concentrated electron beam was projected onto a very thin sample, and the electrons collided with the atoms in the sample and changed direction, thus obtaining an image of the atomic positions and defects. The upper left corner depicts obvious crystal silicon lattice characteristics, whereas the lower right corner shows that the ALD SiO_2_ had no crystal lattice characteristics. After electrons passed through the sample, electrons from the same direction were focused on the same point behind the objective lens, resulting in an electron diffraction pattern. Because the atoms of a crystal have periodic laws, in a crystal material, the diffraction image is composed of a series of regular light spots. Because the atoms of amorphous materials lack the periodic rule of long-range order, the diffraction image turns out to be dispersed concentric circles. The electron diffraction pattern at the interface was obtained via diffraction operation on the ALD SiO_2_, as shown in Figure 1b. Because the electron beam diameter was much larger than the interface width, the diffraction ring formed by the ALD SiO_2_ and the reciprocal lattice formed by the crystal Si could be seen in the electron diffraction pattern.

**Figure 2:**
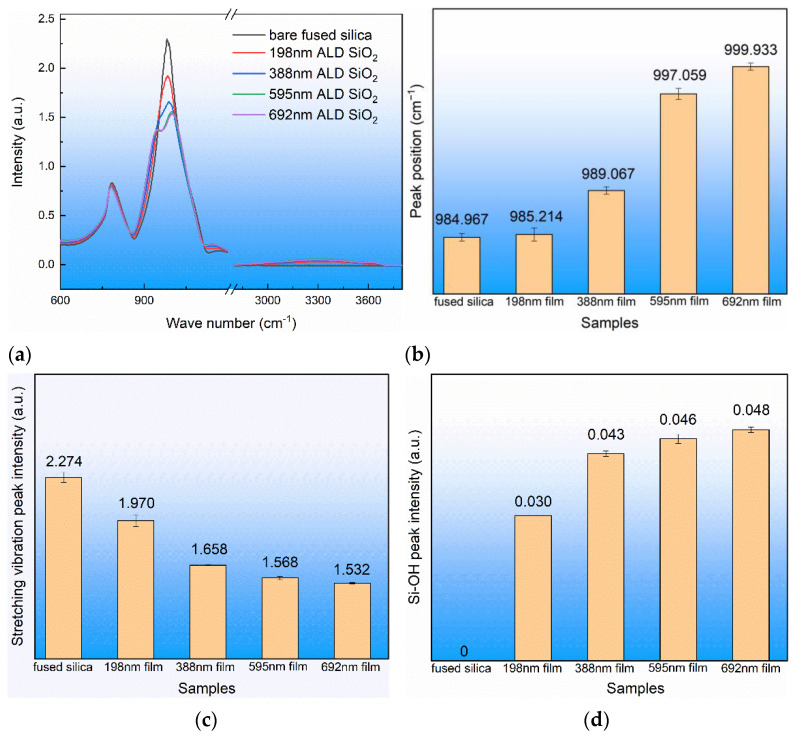
Infrared absorption spectra of ALD SiO_2_ film and fused silica substrate and related vibration peak position and peak intensity. (**a**) Infrared absorption spectra, (**b**) asymmetric stretching vibration peak position of Si-O-Si, (**c**) asymmetric stretching vibration peak intensity of Si-O-Si, and (**d**) Si-OH peak intensity.

The atoms that make up chemical bonds or functional groups are in a state of constant vibration, and their vibration frequency is comparable to that of infrared light. When organic molecules are irradiated with infrared light, the chemical bonds or functional groups in the molecules can be vibrationally absorbed, and different chemical bonds or functional groups have different absorption frequencies, specifically in different absorption peak positions in the infrared spectrum. In this way, the structure of the samples could be obtained by detecting the position of the infrared absorption peak [28,29]. Infrared spectrum tests were carried out on different thicknesses of ALD SiO_2_ film on fused silica substrate, and the results are shown in Figure 2. Each sample was measured at three different locations. The structure of the ALD SiO_2_ film was the same as that of the fused silica substrate, both of which contained a stretching vibration mode and a stretching vibration mode of Si-O. In Figure 2a, the measurement of 980 cm^−1^ is attributed to Si-O asymmetric stretching vibration (Vs), 784 cm^−1^ to Si-O bending vibration (Vb) [30,31], and 950 cm^−1^ and 3300 cm^−1^ to Si-OH vibration [32,33]. It can be seen in Figure 2b that the asymmetric stretching vibration peak position was related to the film thickness and that the vibration peak position gradually increased with the increase in film thickness. It can be seen in Figure 2c that the asymmetric stretching vibration peak intensity was related to the film thickness and that the peak intensity decreased with the increase in film thickness. It can be seen in Figure 2d that the peak intensity of Si-OH increased with the increase in film thickness. It can be judged that the structure of the ALD SiO_2_ film was almost the same as that of the fused silica substrate. The ALD SiO_2_ film contained Si-OH defect and the peak strength of the asymmetric tensile vibration was weaker than that of the fused silica substrate.

**Figure 3:**
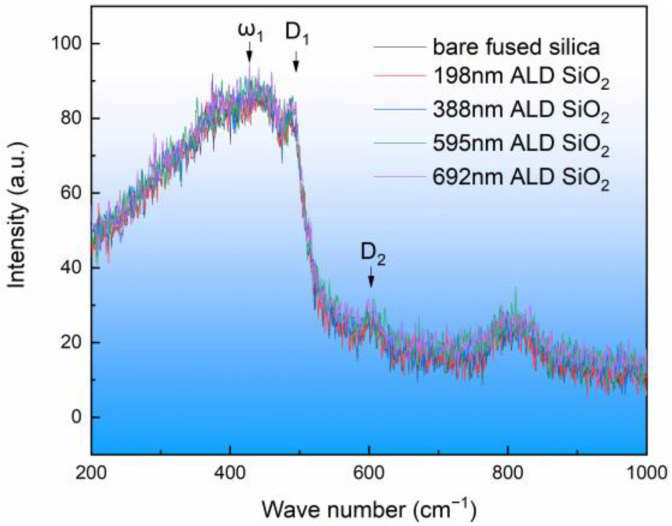
Raman spectra of ALD SiO_2_ film and fused silica substrate.

Raman spectroscopy is an analytical method based on the Raman scattering effect discovered by Indian scientist C.V. Raman. It analyzes the scattering spectra with different frequencies of incident light to obtain information about the molecular vibration and rotation, and it is applied to the study of molecular structure [34]. It is generally believed that fused silica is a spatial network topology composed of a silicon atom and an oxygen atom tetrahedron as the basic unit. In the middle range, silicon and oxygen atoms are connected to each other to form ring structures, such as three-membered rings, four-membered rings, and higher-membered rings. In this experiment, fused silica substrate and ALD SiO_2_ film with different thickness were tested by Raman spectrum, and the test results are shown in Figure 3. Each sample was measured at five different locations. In the Raman spectrum, 440 cm^−1^ (ω_1_) is attributed to oxygen atoms in the ring structure with more than five silicon atom rings, 490 cm^−1^ (D_1_) is attributed to the vibration of oxygen atoms in the four-membered ring, 606 cm^−1^ (D_2_) is attributed to the vibration of oxygen atoms in the three-membered ring, and 800 cm^−1^ is attributed to the bending vibration of Si-O-Si [35,36]. It can be seen in Figure 3 that the peak shape and the peak position of the ALD SiO_2_ film were consistent with those of the fused silica substrate, indicating that the ALD SiO_2_ film and the fused silica substrate had the same structure.

#### 3.1.2. ALD SiO_2_ Film Optical Properties

**Figure 4:**
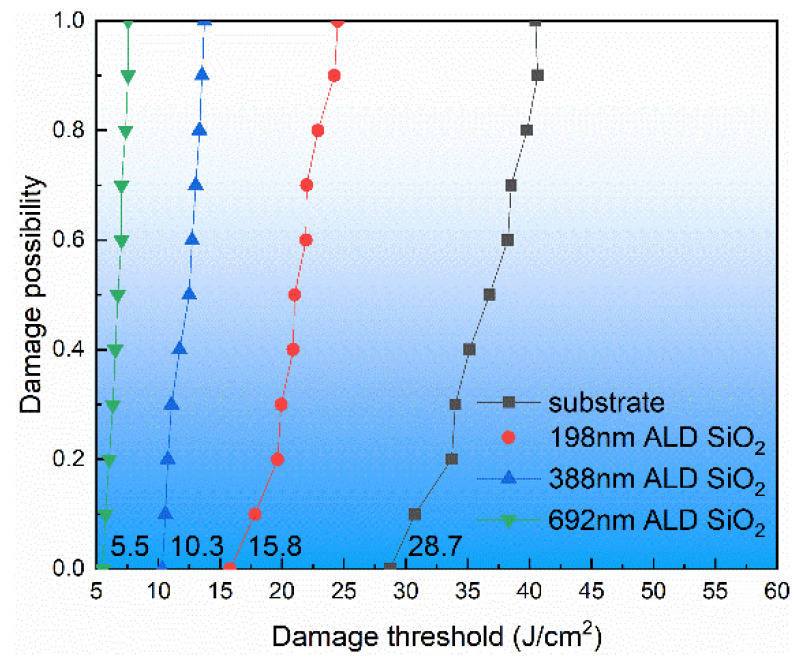
Laser damage threshold of ALD SiO_2_ film and fused silica substrate.

The laser damage threshold is an important parameter for characterizing the ability of a laser irradiated medium to resist laser damage [37]. The damage threshold of the sample in this experiment was obtained with a damage test platform of 355 nm@10 ns. The damage test results for the ALD SiO_2_ film with different thicknesses on the fused silica substrate of the same batch are shown in Figure 4. As can be seen in Figure 4, the 355 nm laser damage thresholds of the fused silica substrate and 198 nm, 388 nm, and 692 nm ALD SiO_2_ were 28.7 J/cm^2^, 15.8 J/cm^2^, 10.3 J/cm^2^, and 5.5 J/cm^2^, respectively. The damage threshold of the ALD SiO_2_ film was much lower than that of the fused silica substrate, and the damage threshold decreased with the increase in film thickness. It can be seen that the film had a greater impact on laser damage than the interface.

#### 3.1.3. ALD SiO_2_ Film Mechanical Properties

**Figure 5:**
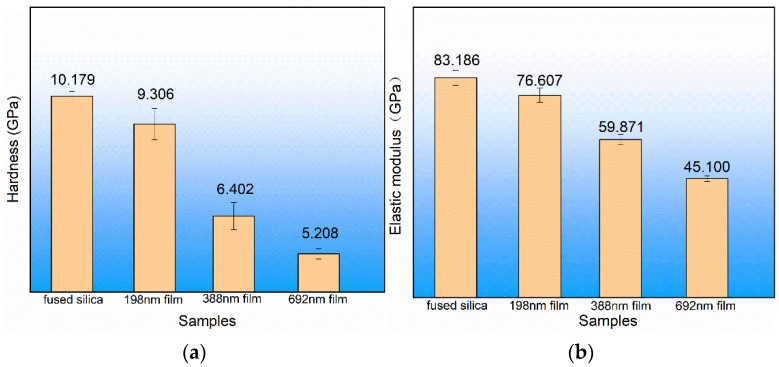
Mechanical properties of ALD SiO_2_ film and fused silica substrate. (**a**) Hardness and (**b**) elastic modulus.

The mechanical properties of a material [38,39], especially the hardness and elastic modulus, directly determine the wear resistance of the material and affects the service life of the material. The hardness and the elastic modulus of the sample in this experiment were obtained with the nano hardness tester. The ALD SiO_2_ film with different thicknesses on the fused silica substrate of the same batch were pressed into a depth of 100 nm for testing, and the results obtained are shown in Figure 5. The same sample was measured in five different regions. It can be seen that the hardness and the elastic modulus of the film were much lower than those of the substrate, and with the increase in film thickness, the hardness and the elastic modulus decreased gradually. This is because as the thickness of the film increased, the measured data were less and less affected by the substrate.

## 4. Effect of Annealing on the Structure and Properties of ALD SiO_2_ Film

### 4.1. Effect of Annealing Temperature on ALD SiO_2_ Film Structure

**Figure 6:**
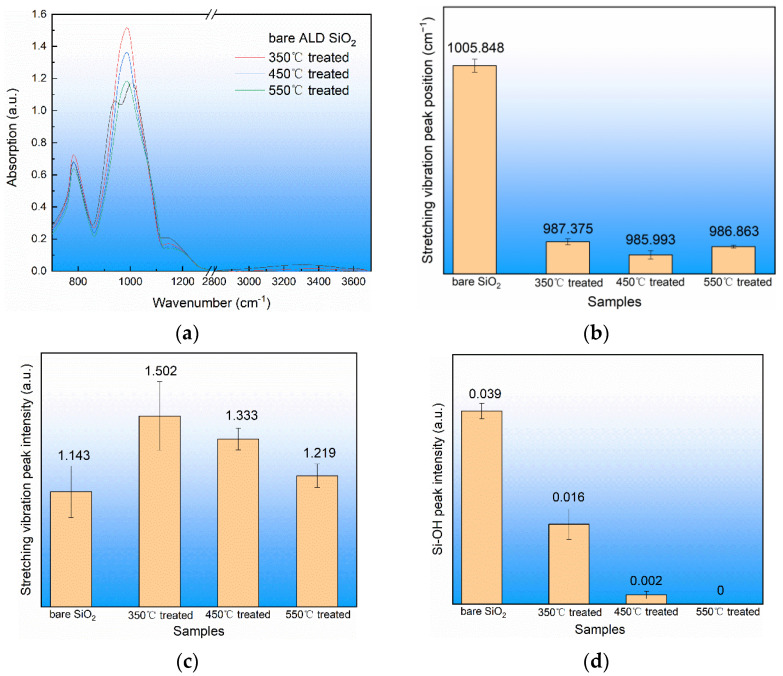
Infrared spectrum of ALD SiO_2_ film before and after thermal annealing. (**a**) Infrared spectrum of ALD SiO_2_ film, (**b**) stretching vibration peak position of Si-O-Si, (**c**) stretching vibration peak intensity of Si-O-Si, and (**d**) Si-OH peak intensity.

In this experiment, the ALD SiO_2_ film on the fused silica substrate of the same batch was used, and then the spectral changes to the ALD SiO_2_ film were measured via infrared reflection. The results are shown in Figure 6. It can be seen in Figure 6a that the waveform changed before and after annealing treatment, indicating that the structure of the ALD SiO_2_ film underwent some changes. It can be seen in Figure 6b that after low-temperature annealing, the peak position of the asymmetric stretching vibration peak of Si-O-Si decreased from the initial 1005.848 cm^−1^ to 985.993 cm^−1^. It can be seen in Figure 6c that after annealing ALD SiO_2_, the peak strength of the asymmetric stretching vibration of Si-O-Si increased from 1.143 to 1.502. It can be seen in Figure 6d that the peak intensity of the Si-OH peak decreased from 0.039 to 0. According to Lamberbier’s law, the content of the asymmetric stretching vibration of Si-O-Si increased after annealing and the content of Si-OH decreased after annealing.

**Figure 7:**
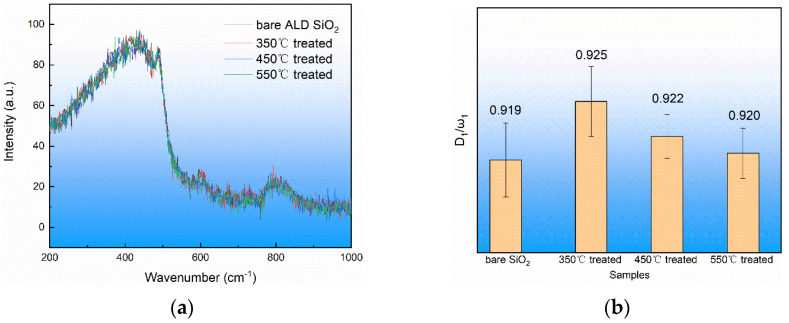
Raman spectra of ALD SiO_2_ film treated at different temperatures. (**a**) Raman spectra of ALD SiO_2_ film and (**b**) the relationship between D_1_/ω_1_ and temperature.

After the ALD SiO_2_ (692 nm) film on the fused silica substrate of the same batch was annealed, the detection was carried out under the Raman spectrometer. The wavelength of the detection laser was 532 nm. The laser was focused onto the surface of the film. In order to reduce the experimental error, five random regions were used for detection. The Raman spectrum was obtained, as shown in Figure 7. It can be seen in Figure 7a that there was no significant change in Raman peak shape and position before or after annealing. In order to reduce the measurement error, the ratio of the peak strength of the four-member ring (D_1_) to the peak strength of main ring (ω_1_) was used in this paper to study the change in the structure of the ALD SiO_2_, and Figure 7b was obtained. It can be seen in Figure 7b that the four-member ring content of the ALD SiO_2_ film increased after annealing.

**Figure 8:**
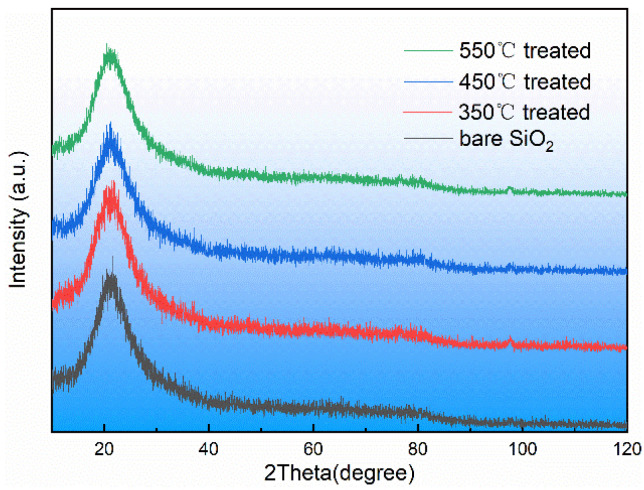
X-ray diffraction spectra of ALD SiO_2_ before and after heat treatment.

X-ray diffraction is especially suitable for phase analysis of crystalline substances. In order to test whether the ALD SiO_2_ crystallized, X-ray diffraction detection of ALD SiO_2_ before and after heat treatment was carried out in this paper, and the detection results are shown in Figure 8. For amorphous materials, because there is no long-range order of atom arrangement in the crystal structure, there is only short-range order in a few atomic ranges, so the XRD pattern of amorphous materials is bulged. It can be seen in Figure 8 that in the whole range of the scanning angle, the scattered X-ray intensity changed gently, during which there was only a maximum value. At the beginning, because the intensity was close to the direct beam, the intensity decreased rapidly with the increase in angle, and the intensity of the high angle slowly tended to the background value of the instrument. The ALD SiO_2_ was amorphous before and after heat treatment, and no crystallization was found. This is because the annealing temperature was much lower than the temperature required for the recrystallization of fused silica.

### 4.2. Effect of Annealing Temperature on Optical Properties of ALD SiO_2_ Film

**Figure 9:**
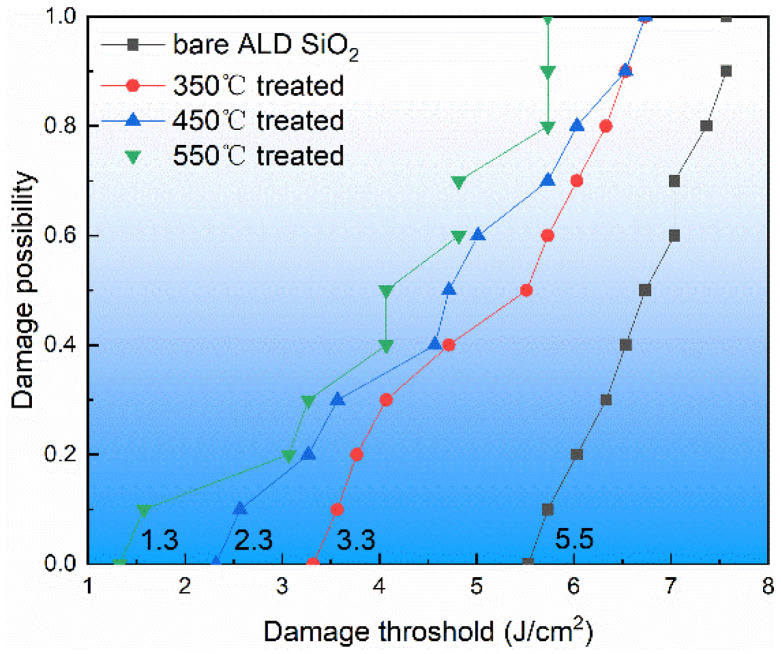
Relationship between ALD SiO_2_ film damage threshold and temperature.

Fused silica is an important component in the laser system, so it is very important to measure its laser damage. When a new component is put into use, the evaluation or determination of its laser damage capability is an essential procedure. The ALD SiO_2_ film of 692 nm thickness on the fused silica substrate of the same batch was irradiated on the damage test platform to obtain the laser damage threshold, and the results are shown in Figure 9. It can be seen in Figure 9 that with the increase in annealing temperature, the damage threshold decreased gradually from the initial 5.5 J/cm^2^ to the lowest point of 1.3 J/cm^2^. In the rapid annealing process, the expansion rate of the film and the substrate was inconsistent, stress between the film and the substrate formed, and the stress led to electronic defects in the film or the expansion of the original electronic defects, thereby reducing the laser damage threshold of the film.

### 4.3. Effect of Annealing Temperature on Mechanical Properties of ALD SiO_2_ Film

**Figure 10:**
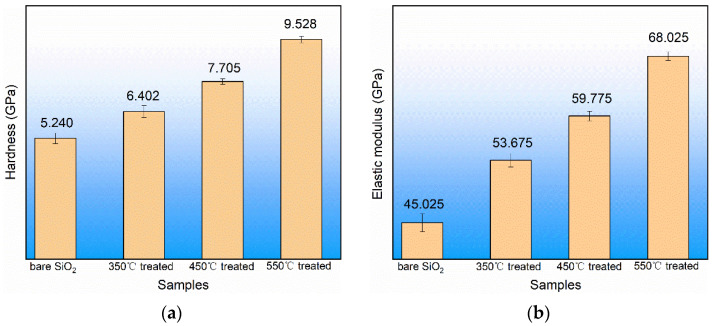
Relation between ALD SiO_2_ mechanical properties and temperature. (**a**) Hardness and (**b**) elastic modulus.

In order to reduce the influence of the equipment and the experimentalists on the experimental results, all samples in this experiment were tested at the same time. A diamond indenter was pressed into the ALD SiO_2_ film (692 nm) at a depth of 100 nm to measure the hardness and the elastic modulus. Each specimen was measured at five different locations and the results were obtained, as shown in Figure 10. It can be seen in Figure 10a,b that after the annealing treatment, the elastic modulus of the ALD SiO_2_ increased from 45.025 GPa to 68.025 GPa and the hardness increased from 5.240 GPa to 9.528 GPa, which is attributed to the increase in four-member ring content causing densification [40], the decrease in Si-OH content, and the increase in the Si-O-Si peak intensity.

## 5. Conclusions

By using BTBAS as an Si precursor and O_3_ as an oxygen precursor, a single layer of ALD SiO_2_ film was deposited on the silicon wafer and on the fused silica substrate, respectively. High-resolution electron microscopy was used to study the ALD SiO_2_ cross-section. The results show that the deposited SiO_2_ had good densification, there were no cavities or other microscopic defects in the internal or interface of the film, and the ALD SiO_2_ coincided with a typical amorphous state. The XRD spectra show that the ALD SiO_2_ did not crystallize after low-temperature annealing. The Raman spectra show that the structure of the ALD SiO_2_ was consistent with that of the fused silica substrate. The infrared spectrum detection shows that the ALD SiO_2_ asymmetric stretching vibration peak position was greater than that of the fused silica substrate, the ALD SiO_2_ asymmetric stretching vibration peak intensity was smaller than that of the fused silica substrate, and at the same time, the ALD SiO_2_ Si-OH peak was greater than that of the fused silica substrate and was proportional to the film thickness. The damage test of the ALD SiO_2_ film on the fused silica substrate shows that the laser damage threshold decreased with the increase in film thickness. The nanoindentation test shows that the hardness and the elastic modulus of the ALD SiO_2_ were much lower than those of the fused silica substrate. After annealing at a low temperature with a rapid warming rate, the ALD SiO_2_ four-member ring content increased, the peak position of the asymmetric stretching vibration of Si-O-Si decreased, the peak Intensity of the asymmetric stretching vibration of Si-O-Si increased, the peak intensity of Si-OH decreased, the laser damage threshold of the ALD SiO_2_ was reduced, the elastic modulus of the ALD SiO_2_ increased from 45.025 GPa to 68.025 GPa, and the hardness increased from 5.240 GPa to 9.528 GPa.

## Data Availability

Data are contained within the article.
